# Pazopanib-Induced Liver Injury in Patients With Metastatic Renal Cell Carcinoma: A Report of Two Cases

**DOI:** 10.7759/cureus.32474

**Published:** 2022-12-13

**Authors:** Ryusuke Ouchi, Shota Kashiwagura, Takashi Watanabe, Kensuke Usui, Jun Ito, Yasuhiro Kaiho, Makoto Sato, Kouji Okada

**Affiliations:** 1 Department of Pharmacy, Tohoku Medical and Pharmaceutical University Hospital, Sendai, JPN; 2 Division of Clinical Pharmaceutics and Pharmacy Practice, Tohoku Medical and Pharmaceutical University, Sendai, JPN; 3 Department of Urology, Tohoku Medical and Pharmaceutical University Hospital, Sendai, JPN

**Keywords:** plasma concentrations, liver injury, renal cell carcinoma, multikinase inhibitor, pazopanib

## Abstract

We report two cases of pazopanib (PAZ)-induced liver injury in patients with metastatic renal cell carcinoma. The first patient was a 70-year-old female who was diagnosed with right renal cell carcinoma and showed tumor embolism in the inferior vena cava. PAZ was started but discontinued after about one month due to a grade four liver injury. The second patient was a 60-year-old male who was diagnosed with left renal cell carcinoma and suspected multiple lung metastases. PAZ was started following a laparoscopic left radical nephrectomy but was stopped after about a month due to a grade three liver injury. We analyzed the plasma PAZ concentrations for treatment evaluation. High plasma PAZ concentrations were observed in both patients after PAZ treatment began. Severe liver injury after PAZ administration may be associated with high plasma PAZ concentrations; hence, we should reduce PAZ dosage early. We also recommend monitoring plasma PAZ concentrations, if possible, so that physicians can either reduce the dosage or discontinue treatment to avoid further liver damage.

## Introduction

The multikinase inhibitor pazopanib (PAZ), which exerts its antitumor effects by targeting several kinases, is used to treat metastatic renal cell carcinoma by inhibiting interstitial intracellular signaling [1]. Generally, oral molecular-targeted therapies exhibit large differences in pharmacokinetics among patients, and poor adherence is a risk factor for treatment failure. The possibility of personalized administration of PAZ has been suggested [2], and patients with renal cell carcinoma whose trough plasma PAZ concentrations exceed 20.5 μg/mL show prolonged progression-free survival [3]. However, fatal liver injury, cardiac injury, and thromboembolic events have been reported as serious adverse effects of PAZ [4]. The U.S. Food and Drug Administration recommends reducing the dose or discontinuing administration depending on the severity of liver injury [5]. Additionally, PAZ is assigned score C (probable cause of clinically apparent liver injury) in the LiverTox database [6]. Although there are several reports of liver injury due to PAZ [7,8], to the best of our knowledge, there are no case reports regarding pazopanib-induced liver injury in patients with metastatic renal cell carcinoma that investigated plasma concentrations. Here, we present two cases of liver injury during PAZ therapy with increased plasma PAZ concentrations in patients with renal cell carcinoma.

## Case presentation

Case one

**Figure 1:**
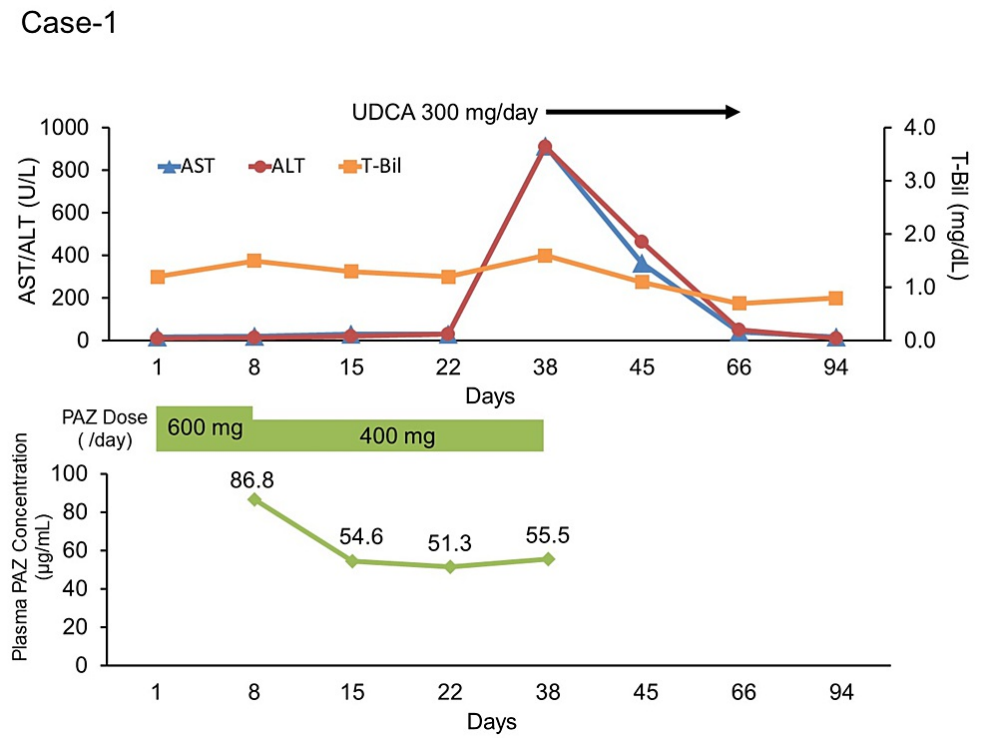
Transition in pazopanib dose, plasma PAZ concentration, and laboratory data in case one Day one represents the start date of pazopanib treatment. ALT: alanine aminotransferase; AST: aspartate aminotransferase; PAZ: pazopanib; T-Bil: total bilirubin; UDCA: ursodeoxycholic acid

A 70-year-old female was diagnosed with right renal cell carcinoma and showed tumor embolism in the inferior vena cava. She had a history of hypertension, hyperlipidemia, and reflux esophagitis. Her records indicated that she was taking the following tablets: amlodipine besylate, 10 mg/day; azilsartan, 10 mg/day; simvastatin, 5 mg/day; alprazolam, 0.4 mg/day; vonoprazan fumarate, 20 mg/day; and zolpidem tartrate, 5 mg/day. Upon examination, we planned a laparoscopic right radical nephrectomy but later opted for PAZ administration because of the tumor embolism. The patient was hospitalized to start PAZ under medical supervision and administered PAZ 600 mg/day (days one to seven). Vonoprazan fumarate and simvastatin were discontinued at the time of hospitalization because they could result in decreased PAZ absorption and liver injury, respectively. On day eight, the patient complained of dizziness, and her blood pressure was high (163/81 mmHg). Therefore, the dose of PAZ was reduced to 400 mg per day. After the discontinuation of vonoprazan fumarate, the patient complained of heartburn at night. Therefore, famotidine 20 mg/day, which has a shorter half-life than that of vonoprazan fumarate, was administered once before bedtime on day 15. Thereafter, the patient’s general condition stabilized, and PAZ administration was continued at the same dose. The patient was discharged on day 22, but treatment was continued at the outpatient department. On day 38, the patient complained of fatigue. Furthermore, aspartate aminotransferase (AST) and alanine transaminase (ALT) levels were 912 U/L (grade four) and 900 U/L (grade four), respectively. Therefore, PAZ was discontinued, and ursodeoxycholic acid (UDCA) at 300 mg/day was started to ameliorate liver function. On day 66, the ALT level decreased to 40 U/L, and the ALT level decreased to 59 U/L (both grade 1), so we stopped UDCA. However, on day 94, PAZ was replaced with axitinib at the request of the patient. The plasma PAZ concentrations on days 8, 15, 22, and 38 were 86.8, 54.6, 51.3, and 55.5 µg/mL, respectively (Fig. 1). Adverse effects were evaluated using the Common Terminology Criteria for Adverse Events (CTCAE) version 5.0. The trough plasma PAZ concentration was measured using liquid chromatography with tandem mass spectrometry (LC-MS/MS).

Case two

**Figure 2:**
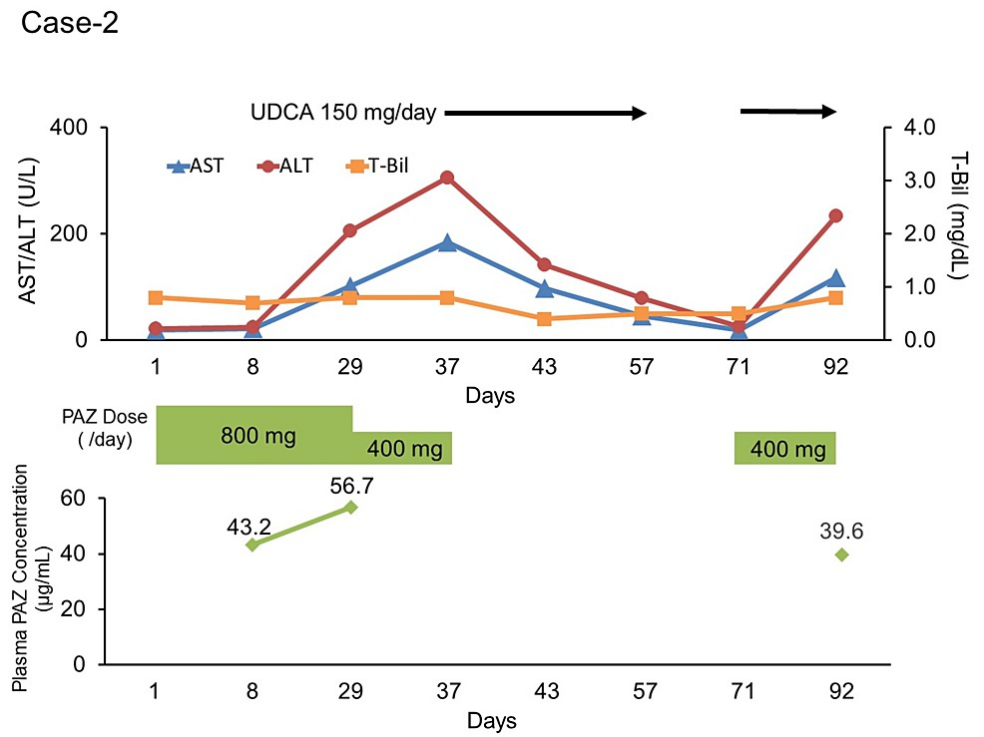
Transition in pazopanib dose, plasma PAZ concentration, and laboratory data in case two Day one represents the start date of pazopanib treatment. ALT: alanine aminotransferase; AST: aspartate aminotransferase; PAZ: pazopanib; T-Bil: total bilirubin; UDCA: ursodeoxycholic acid

A 60-year-old male was diagnosed with left renal cell carcinoma and suspected multiple lung metastases. He had a history of chronic obstructive pulmonary disease, diabetes mellitus, hypertension, and congenital heart disease. His records indicated that he was taking the following tablets: sitagliptin phosphate hydrate, 50 mg/day; metformin hydrochloride, 1500 mg/day; telmisartan, 80 mg/day; and amlodipine besylate, 10 mg/day. Laparoscopic left radical nephrectomy was performed for left renal cell carcinoma, but postoperative CT scans showed multiple lung tumors; a biopsy revealed lung metastasis. Therefore, PAZ at 800 mg/day was administered in the outpatient department (on day one). On day 29, AST and ALT levels were 102 U/L (grade one) and 205 U/L (grade three), respectively. Therefore, PAZ was reduced to 400 mg per day. On day 37, the patient was diagnosed with gout at another hospital and was informed of liver injury following a blood test. When another blood test was performed at our hospital, an increase in AST (184 U/L) and ALT levels (305 U/L) (both grade 3) was observed, and the patient was diagnosed with PAZ-associated liver injury. Therefore, PAZ was withdrawn and UDCA 150 mg/day was started to ameliorate liver function. UDCA was discontinued on day 57 owing to a decrease in the AST (46 U/L; grade one) and ALT (79 U/L; grade one) levels. On day 71, the AST and ALT levels improved to 19 U/L and 25 U/L, respectively. Subsequently, PAZ 400 mg/day was resumed in combination with UDCA 150 mg/day on day 85. However, on day 92, the AST and ALT levels increased again to 117 U/L (grade 2) and 233 U/L (grade 3), respectively. Therefore, the administration of PAZ was discontinued. Of note, the plasma PAZ concentrations on days 8, 29, and 92 were 43.2, 56.7, and 39.6 µg/mL, respectively (Fig. 2). Adverse effects were evaluated using CTCAE version 5.0. The trough plasma PAZ concentration was measured using LC-MS/MS.

## Discussion

We came across two cases of severe PAZ-induced liver injury, which developed after PAZ administration and was alleviated after PAZ discontinuation. In a meta-analysis of abnormal liver function tests, it was estimated that the median time for the onset of liver injury due to PAZ treatment in patients with renal cell carcinoma, malignant soft tissue sarcoma, and ovarian cancer [shown as ALT > 3.0× the upper limit of normal (ULN)] was 42 (20-182) days. The median time from onset to recovery was 30 (7-168) days [9]. The patients we came across followed a similar course. The Roussel-Uclaf Causality Assessment Method (RUCAM) scale [10] is an established tool commonly used to quantitatively assess causality in cases of suspected drug-induced liver injury (DILI). The RUCAM scale is based on time to onset from the start of the drug, course of ALT after drug discontinuation, risk factors, and concomitant drug. Subsequently, based on the total score, the probability of DILI will be assessed as follows: ≤0, excluded; one to two, unlikely; three to five, possible; six to eight, probable; and ≥ nine, highly probable. The RUCAM scores for cases one and two were six (probable) and 10 (highly probable), respectively. Notably, liver metastasis was not observed in both cases, and no other liver disease was observed. Therefore, this suggested that liver injury was related to PAZ administration in both cases. 

With respect to the relationship between plasma PAZ concentrations and toxicity, it has been reported that the incidence of grade three or higher fatigue, anorexia, and hypertension increases when the plasma PAZ concentrations exceed 50.3 µg/mL [11]. Furthermore, PAZ is more toxic when plasma PAZ concentrations exceed 46 µg/mL [3,12]. The plasma PAZ concentration threshold for severe liver injury is not well-defined, but plasma PAZ concentrations in our patients were above the threshold for serious adverse events reported in a previous study [3,11,12]. A study on Japanese patients investigating dose optimization based on plasma PAZ concentrations reported that a dose of 400 mg/day was efficacious and safe for more than half of the patients [13]. Even though the exact cause of the high plasma PAZ concentrations in our two patients-despite being started at normal or low doses-is unknown, based on reported literature, we should have reduced the starting PAZ doses in both patients. Grade three or higher liver injury has been reported even with low plasma PAZ concentrations in very few cases [14]. However, from our experience, we assume that PAZ-induced liver injury is related to elevated plasma PAZ concentrations. Furthermore, our two patients had high plasma PAZ concentrations before the onset of liver injury; therefore, we consider that high plasma PAZ concentrations in the early stages of treatment may be a risk factor for the onset of PAZ-induced liver injury. Although CYP3A4 inhibitors were not used concomitantly in our cases, we should pay attention to the increase in plasma pazopanib concentrations if CYP3A4 inhibitors are used concomitantly [5].

Plasma PAZ concentrations in our patients were measured later in the research framework rather than immediately when the patient was undergoing treatment. However, plasma PAZ concentrations are not measured in routine clinical practice. In Japan, the measurement of plasma PAZ concentrations is not covered under medical insurance. Periodic liver function tests are recommended for the management of PAZ-induced liver injury [5], but this alone is insufficient. We believe that plasma PAZ concentration-guided monitoring further ensures the prevention of PAZ-induced liver injury and, therefore, should be implemented in clinical practice. If plasma PAZ concentrations are excessively elevated at the beginning of treatment, we can consider early dose reduction or switching to axitinib as a treatment option to prevent adverse events such as serious liver injury.

## Conclusions

PAZ administration for renal cell carcinoma treatment is associated with severe liver injury, particularly in patients with high plasma PAZ concentrations in the early stage of treatment, and can restrict further treatment. Plasma PAZ concentration-guided management may result in PAZ dose reduction in the early stages of cancer treatment to avoid severe PAZ-induced liver injury.
